# Patients with infective endocarditis undergoing cardiac surgery have distinct ROTEM profiles and more bleeding complications compared to patients without infective endocarditis

**DOI:** 10.1371/journal.pone.0284329

**Published:** 2023-04-13

**Authors:** Jennifer S. Breel, Agnes G. C. L. Wensing, Susanne Eberl, Benedikt Preckel, Patrick Schober, Marcella C. A. Müller, Robert J. M. Klautz, Markus W. Hollmann, Henning Hermanns

**Affiliations:** 1 Department of Anaesthesiology, Amsterdam University Medical Centres, Amsterdam, the Netherlands; 2 Department of Intensive Care, Amsterdam University Medical Centres, Amsterdam, the Netherlands; 3 Department of Cardiac Surgery, Amsterdam University Medical Centres, Amsterdam, the Netherlands; BSMMU: Bangabandhu Sheikh Mujib Medical University, BANGLADESH

## Abstract

**Background:**

The coagulation system is crucial in the pathogenesis of infective endocarditis and undergoes significant changes during course of the disease. However, little is known about the implications of those changes in the perioperative period. Aim of the present study was to delineate the specific coagulation patterns and their clinical consequence in patients undergoing cardiac surgery due to infective endocarditis.

**Methods:**

In this single-centre, exploratory, prospective observational study, we investigated the incidence and degree of coagulopathy in patients with (n = 31) and without infective endocarditis (n = 39) undergoing cardiac valve surgery. The primary outcome was the differences between these two groups in rotational thromboelastometry (ROTEM) results before, during and after surgery. The secondary outcomes were the differences between the groups in heparin sensitivity, bleeding complications, and transfusion requirements.

**Results:**

Most ROTEM parameters in EXTEM, INTEM and FIBTEM assays were significantly altered in patients with infective endocarditis. Clotting time in the EXTEM assay was significantly prolonged in the endocarditis group at all time-points, while all clot firmness parameters (A5, A10 and MCF) were significantly increased. The heparin sensitivity index was significantly lower in the endocarditis group (median index 0.99 vs 1.17s. IU^-1^.kg^-1^, p = .008), indicating increased heparin resistance. Patients with infective endocarditis had more bleeding complications as assessed by the universal definition of perioperative bleeding score (OR 3.0, p = .018), and more patients with endocarditis underwent early re-exploration (p = .018).

**Conclusions:**

The findings of this exploratory investigation show significantly altered coagulation profiles in patients with infective endocarditis, with concomitant hyper- and hypocoagulability. Furthermore, the incidence of bleeding complications and transfusion requirements were increased in patients with endocarditis. These results show the potential of ROTEM to detect coagulation abnormalities in patients with infective endocarditis. Existing point-of-care coagulation testing guided algorithms for optimizing perioperative coagulation management possibly need to be adjusted for these high-risk patients undergoing cardiac surgery.

## Introduction

Infective endocarditis (IE) is a multisystem disease caused by an infection (usually bacterial) of the cardiac endothelium [1]. The incidence of IE is approximately 3–15 per 100 000 people and is steadily increasing, due to longevity, the increased number of invasive cardiac procedures and health-care related infections [2].

The mortality of IE is high, with one third of all patients dying within one year. Major complications are seen in up to 60% of patients with IE, of which the most common are stroke, non-stroke embolisation, intracardiac abscess formation and heart failure. Acute heart failure due to valvular dysfunction requires (urgent) surgical intervention, and in case of cardiogenic shock emergency surgery is indicated [3].

The coagulation system is profoundly activated in IE. Initiation and progression of an endocarditis vegetation as well as thromboembolic events are possible consequences of these perturbations. The common activation pathways and feedback mechanisms serve as triggers for both, coagulation and inflammation, which appear to play a crucial role in the development and persistence of IE [4].

Patients with active IE are mostly in a hypercoagulable state. Thrombin generation is enhanced, with concomitantly reduced activity of the anticoagulant and fibrinolytic system. Ultimately, thrombin activates platelets and converts fibrinogen into fibrin strands, forming a blood clot [4, 5]. This condition escalates IE vegetation maturation and increases the risk of thromboembolic events. In severe cases of IE, disseminated intravascular coagulation (DIC) may occur. DIC significantly augments the need for perioperative blood product transfusion in patients with IE, and increases the risk of multi-organ failure and mortality [6]. The precise mechanisms underlying the hypercoagulable state in patients with IE are yet unclear, but hypercoagulation is further amplified by ongoing inflammation, sepsis, and organ dysfunction [7].

Patients with IE undergoing multi-valve surgery for eradication of perivalvular infection often require longer aortic cross-clamping and cardiopulmonary bypass (CPB) time, which additionally increase the risk for coagulopathy, transfusion of blood products, and mortality [8].

Intravenous heparin administration is the gold standard of intraoperative anticoagulation to prevent clot formation during CPB. The intraoperative anti-coagulant effect of heparin is controlled using activated clotting time (ACT). Corresponding to their hypercoagulable state, patients with IE show a decreased heparin sensitivity, and therefore frequently require higher doses of heparin to reach adequate prolongation of ACT [9].

Based on the presented changes of the coagulation system, management of coagulation disturbances and bleeding complications represent particular challenges in patients with IE [10]. Intraoperative point-of-care coagulation testing (POCT), such as rotational thromboelastometry (ROTEM) may assist in decision-making concerning perioperative coagulation management [11]. While in other systemic diseases that significantly affect the coagulation system, such as sepsis and Covid-19, ROTEM has emerged as promising tool in diagnosing alterations in coagulation [12, 13], there is no evidence for its use in IE.

The primary aim of this study was to determine the incidence and degree of perioperative dysregulation of coagulation in patients with IE vs. patients undergoing cardiac valve surgery for other reasons, measured by ROTEM. These results will be valuable for future studies investigating the clinical benefit of ROTEM guided treatments in IE. Secondary aims included the evaluation of other factors associated with coagulopathy in patients with IE, compared to patients undergoing valve surgery without IE, such as heparin sensitivity, bleeding complications and transfusion requirements.

## Methods

This single-centre exploratory prospective observational study was performed in the Amsterdam UMC, location AMC, and collected data from patients undergoing cardiac valve surgery from 21 January 2020 until 15 October 2020. The Ethical Committee of AMC confirmed that the study did not fall under the Medical Research with Human Subjects Act (WMO) (W19_443 # 19.513). The study adhered to the principles of the Declaration of Helsinki (Fortaleza), Good Clinical Practice and the European Privacy Act (GDPR). A member of the study team approached potential participants, to obtain written informed consent. Inclusion criterion was cardiac valve surgery in adult patients with or without IE. All consecutive surgically treated patients with IE within the study period were approached. Patients without IE, and scheduled for valve surgery, were randomly approached in the same week for the control group. Exclusion criteria were an inability to sign informed consent and a history of hereditary coagulation disorders. Study data were collected from the electronic health record (EPIC Systems Corporation, Verona, WI, USA) and the ROTEM Delta machine (Tem Innovations, Germany). Treatment with blood products and coagulation factors was at the discretion of the attending cardiac anaesthetist, following the department’s ROTEM-guided coagulation management algorithm.

*Primary outcome*: The difference in ROTEM parameters between groups.

ROTEM analysis was performed directly before surgery (after induction of anesthesia), during surgery (after removal of the aortic cross-clamp and before protamine administration), and directly after surgery, before transferring the patient to the Intensive Care Unit.

For each measurement, 2.7 ml of arterial blood was drawn and collected in a citrated tube. The ROTEM delta machine uses electronic pipetting of the reagents with the blood sample and performs analysis in *real-time*. The machine produces 20 different parameters, eight of which were used for analysis: Clotting time (CT), amplitude 5 and 10 minutes after clotting time (A5 and A10), maximum clot firmness (MCF), clot formation time (CFT) and alpha angle (the angle of the tangent between clot firmness 0 and 20 mm, in degrees), maximum clot elasticity (MCE) and area under the curve (AUC) [14]. In a few patients, at some time points, one or more channels showed erroneous measurements, e.g. due to pipetting mistakes. Those measurements were excluded from analysis, which explains some measurements presented only contain 31 or 39 measurements, respectively.

*Secondary outcome*: The difference in heparin sensitivity, bleeding complications, and transfusion requirements between groups.

Heparin resistance was defined as the inability to achieve an ACT of at least 450 seconds with an adequate initial dose of heparin (300 IU.kg^-1^) [15]. The heparin sensitivity index (HSI) [16] was calculated and compared between both groups as:

$$HSI=\frac{ACT\;after\;initial\;heparin\;dose\;(s)-Baseline\;ACT(s)}{Initial\;dose\;of\;heparin\;(IU\;per\;kg\;body\;weight)}$$

Bleeding complications were assessed using the universal definition for perioperative bleeding (UDPB), which uses nine events occurring intra- or postoperatively: delayed sternal closure, postoperative chest tube output, transfusion of packed red blood cells (RBC), fresh frozen plasma (FFP) or thrombocytes, administration of cryoprecipitate or fibrinogen concentrate, the use of factor concentrates and recombinant activated factor VII (rFVIIa) as well as surgical re-exploration. The five classes are designed to characterize the severity of bleeding, regardless of the source: Class 0 (insignificant), Class 1 (mild), Class 2 (moderate), Class 3 (severe), Class 4 (massive) [17].

### Statistics

Coded data were entered into a GCP compliant database after collection (Castor EDC, Garden City, NY, United States). All data entries were double-checked by a second member of the study team. Data analysis was done using IBM SPSS v.26 and R (4.0.3). No sample size was calculated in this exploratory investigation. Descriptive statistics were used to present patient characteristics. All data were tested for accuracy, missing data, outliers, and heteroscedasticity. Means with standard deviations or medians with minimum-maximum were reported to show the full range of the dataset, including outliers. Categorical and binary data were presented as absolute frequencies with percentages. Unpaired numerical ROTEM data in the IE and non-IE groups were tested for differences in distribution using the Mann Whitney U test. Statistical significance was assumed at p < .05.

## Results

**Fig 1:**
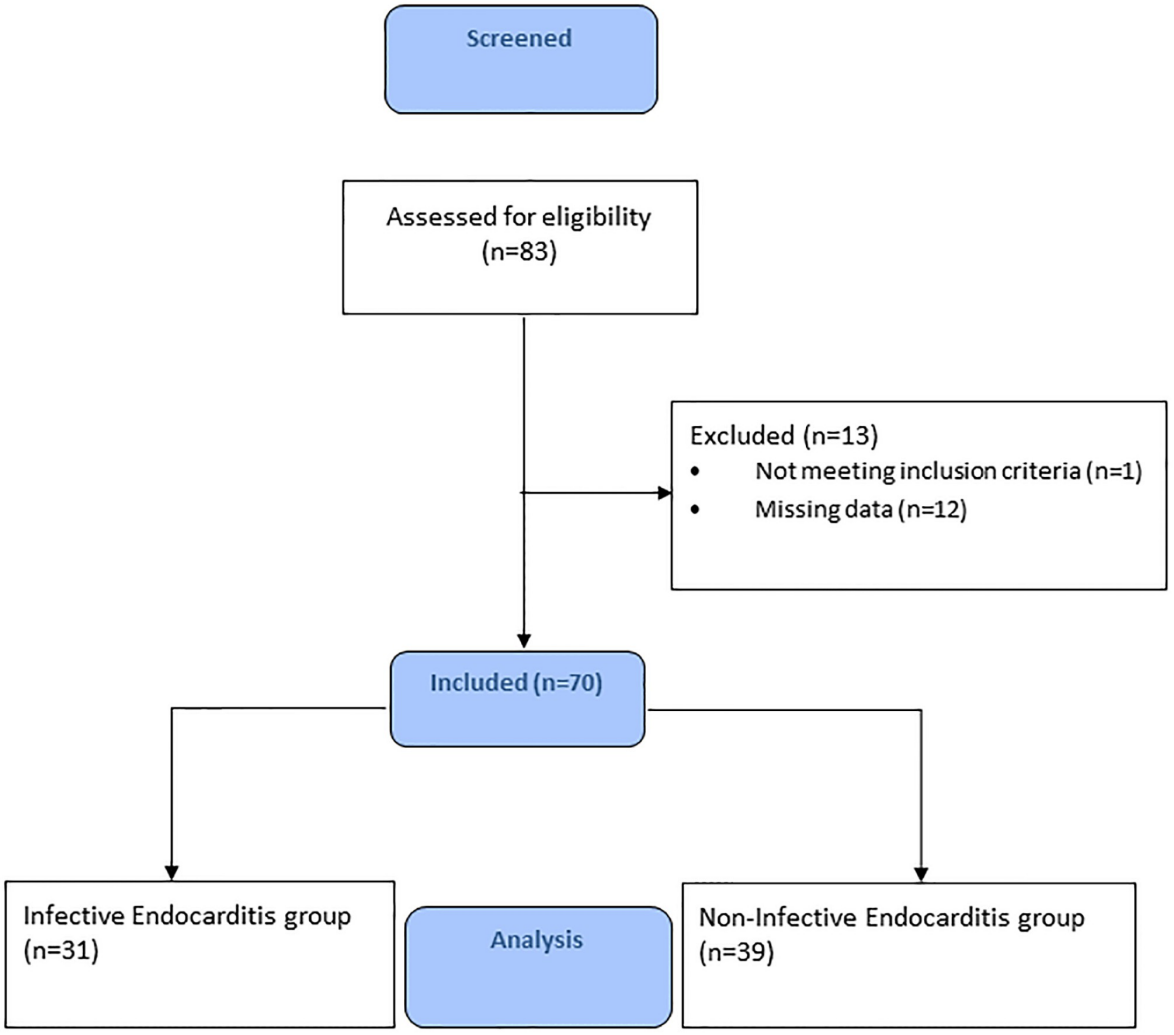
Flowchart of screening and inclusion.

We screened 83 patients of which 70 were included, 31 in the IE group and 39 in the non-IE group (Fig 1). The median sternotomy approach was used in all patients. Preoperative patient characteristics are shown in Table 1. The infective agents in the IE group can be found in S4 Table. Compared to patients undergoing elective surgery, patients with IE were predominantly male 25 (81%), with a median (mdn) age of 67 years (min-max 29–84), a lower body mass index (mdn 24 kg.m^-2^ (min-max 18–31) vs 27 kg.m^-2^ (17–41), *p* = .006), a higher ASA classification (10 vs 3 patients with ASA 4, *p* = .010) and a higher EuroSCORE II: mdn 15.5% (IQR 4.5–34.1) vs 5.4% (IQR 2.7–8.8). More patients with IE had preoperative complications (20 vs 7, *p* < .001). One patient with IE developed overt disseminated intravascular coagulation (DIC) before surgery and received preoperative thrombocyte transfusion. The was no history of IV drug use in either group. All operations in patients with IE were classified as urgent surgery, vs none of the patients in the control group. Cardiopulmonary bypass time and aortic cross clamp time were not different between the groups.

**Table 1:** Characteristics of patients with and without infective endocarditis.

Variable	All patients (N = 70)	Endocarditis (n = 31)	Non-Endocarditis (n = 39)	p-value
Sex				.085
Male n (%)	49 (70)	25 (81)	24 (62)	
Female n (%)	21(30)	6 (19)	15 (28)	
Age, years	67 (21–84)	67 (29–84)	65 (21–81)	.287
Height, cm	176 (156–193)	174 (156–191)	181 (162–193)	.025
Weight, kg	79 (48–148)	78 (57–104)	80 (48–148)	.372
BMI kg.m^-2^	26 (17–41)	24 (18–31)	27 (17–41)	**.006**
ASA classification:				.010
II	4 (6)	1 (3)	3 (8)	
III	53 (75)	20 (65)	33 (85)	
IV	13 (19)	10 (32)	3 (7)	
EuroSCORE II	6.6 (83.5)	15.3 (1–83.5)	5.3 (1–36.5)	**.002**
Diabetes	8 (11)	4 (13)	4 (10)	.731
Previous cardiac surgery (yes) n (%)	29 (41)	16 (52)	13 (33)	.126
CIED	6	3	3	.770
Anticoagulant therapy	49 (70)	23 (74)	26 (67)	.498
Pre-operative complications:	27	20	7	**< .001**
Non-stroke emboli	3	2	1	.839
Venous thrombosis	3	3	0	.287
Ischaemic stroke	5	5	0	.152
Type of surgery				.214
Single valve	19 (27)	9 (29)	10 (26)	
Double valve	6 (9)	2 (6)	4 (10)	
Single valve + other surgery	35 (50)	11 (35)	24 (62)	
Double valve + other surgery	10 (14)	9 (29)	1 (2)	

All values are median (min-max), all p-values were calculated with the Mann Whitney U test, significant values in bold. Abbreviations: IE = Infective Endocarditis, BMI = Body Mass Index, ASA = American Society of Anaesthesiologists, CIED = cardiac implantable electronic device, min-max = minimum-maximum.

Differences in classical laboratory values are presented in Table 2. Patients with IE had a significantly lower haemoglobin (median (min-max); 108 g.l^-1^ (81–147) vs 139 (105–161, *p* < .001), and a higher C-reactive protein (CRP): 46 mg.l^-1^ (0,6–151) vs 12 (0,3–139), *p* < .001). No patients died within 30 days in either group. At one year, three patients (10%) in the IE group had died and one (3%) in the non-IE group.

**Table 2:** Preoperative laboratory values in patients with and without infective endocarditis.

Variable	All patients (N = 70)	Endocarditis (n = 31)	Non-Endocarditis (n = 39)	p-value
				
Haemoglobin (g.l^-1^)	129 (81–161)	108 (81–147)	139 (105–161)	**< .001**
Platelet count (10^9^.l^-1^) n = 65	248 (21–420)	250 (21–420)	225 (110–336)	.232
aPTT (s) n = 21	26 (21–65)	27 (21–65)	26 (23–39)	.856
PT (s) n = 19	12 (11–22)	13 (11–22)	11 (11–20)	.116
INR n = 43	1.1 (0.9–2.9)	1.2 (1–2.4)	1.1 (0.9–2.9)	.078
CRP (mg.l^-1^)	15 (0.3–151)	46 (0.6–151)	12 (0.3–139)	**< .001**
Creatinine (g.l^-1^)	92 (46–272)	104 (46–272)	86 (59–240)	.170

All values are median (min-max), all p-values were calculated with the Mann Whitney U test, significant values in bold. Abbreviations: IE = Infective Endocarditis, aPTT = Activated Partial Thromboplastin Time, PT = prothrombin Time, INR = International Normalized Ratio, CRP = C-reactive Protein

**Fig 2:**
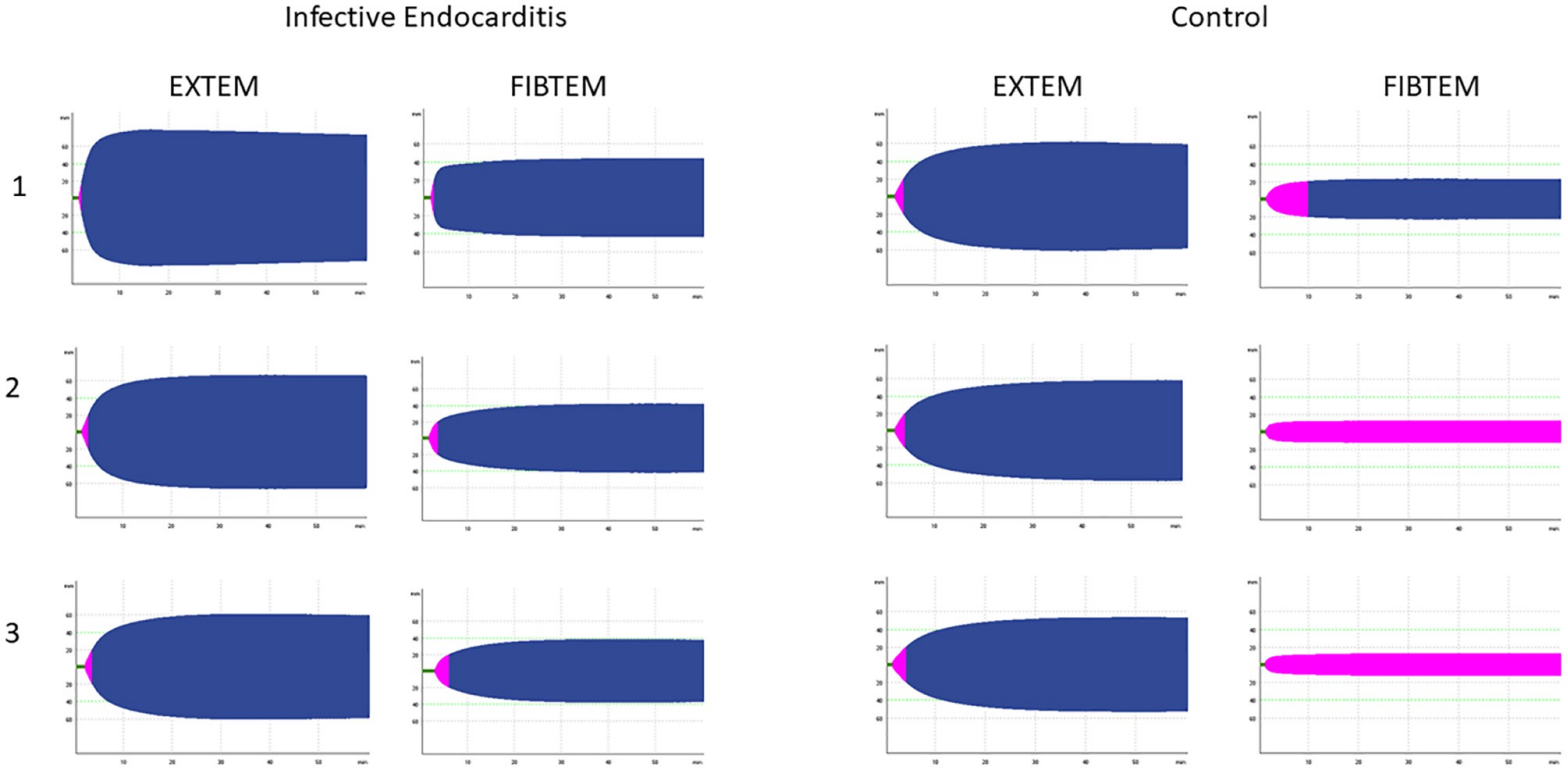
Exemplary TEMograms of ROTEM channels EXTEM and FIBTEM. TEMograms shown from patients with and without infective endocarditis at three time points: 1. after induction of anesthesia, 2. directly after removal of the aortic cross-clamp before protamine administration, and 3. at the end of surgery.

ROTEM parameters before start of surgery showed that nearly all variables of the EXTEM, INTEM and FIBTEM assays were significantly different between the groups: while CT EXTEM was significantly prolonged in patients with IE, all clot firmness parameters were increased when compared to patients without IE (Fig 2 and Table 3). The same pattern was observed after aortic declamping and, to a lesser extent at the end of surgery (S1 and S2 Tables). In contrast, lysis parameters were not significantly different between groups at any time point (S3 Table).

**Table 3:** Preoperative ROTEM parameters of patients with and without infective endocarditis.

ROTEM parameter	Endocarditis (n = 31)	Non-Endocarditis (n = 39)	*p-value*
**EXTEM**	*n = 28*	*n = 39*	
CT (s)	72 (56–123)	62 (38–113)	**.004**
A5 (mm)	55 (39–70)	49 (31–61)	**< .001**
A10 (mm)	64 (49–77)	59 (40–69)	**< .001**
CFT (s)	60 (36–104)	73 (50–148)	**< .001**
MCF (mm)	70 (58–83)	67 (51–76)	**.003**
α (degrees)	78 (74–83)	75 (65–82)	**< .001**
MCE	235 (139–474)	200 (104–316)	**.004**
AUC	6950 (5727–8175)	6542 (8–7471)	**.001**
**INTEM**	*n = 30*	*n = 38*	
CT (s)	184 (114–313)	180 (122–303)	.566
A5 (mm)	55 (35–76)	48 (25–58)	**< .001**
A10 (mm)	64 (48–81)	57.5 (37–67)	**< .001**
CFT (s)	57 (28–144)	67 (40–181)	**.006**
MCF (mm)	70 (54–83)	64 (49–73)	**.001**
α (degrees)	79 (66–84)	77 (58–82)	**.004**
MCE	233 (120–493)	176 (94–276)	**.002**
AUC	6925 (5464–8213)	6344 (4682–7250)	**< .001**
**FIBTEM**	*n = 29*	*n = 39*	
CT (s)	69 (51–95)	58 (44–90)	**.001**
A5 (mm)	25 (15–44)	15.5 (5–32)	**< .001**
A10 (mm)	28 (16–47)	17 (6–33)	**< .001**
CFT (s)	103 (34–1326)	171 (4–1115)	.097
MCF (mm)	32 (18–51)	18 (6–36)	**< .001**
α (degrees)	79 (68–84)	76 (61–84)	**.012**
MCE	46 (22–104)	22 (6–55)	**< .001**
AUC	3114 (1740–5051)	1804 (560–3507)	**< .001**

All values are shown as median (min-max), all tests show Mann Whitney U distribution testing, significant values in bold, n = number of patients with valid observations. Abbreviations: s = seconds, CT = clotting time, A5 = amplitude at 5 minutes, A10 = amplitude at 10 minutes, CFT = clot formation time, MCF = maximum clot formation, α = alpha angle, MCE = maximum clot elasticity, AUC = area under the first derivate curve. The CT and CFT are measured in seconds, alpha-angle is measured in degrees, MCF and A10 are measured in mm.

Generally, clot firmness parameters were lower after declamping when compared to preoperative values. Concerning FIBTEM, when comparing preoperative and post-declamping measurements, clot firmness parameters decrease significantly more in patients with IE (Table 4), reflecting a proportionally greater loss of fibrinogen during surgery.

**Table 4:** Differences between preoperative and post-declamping FIBTEM in patients with and without infective endocarditis.

Δ FIBTEM 1—FIBTEM 2			
	Endocarditis (*n = 29)*	Non-Endocarditis *(n = 38)*	p-value
A5 (mm)	10 (-22; 20)	4 (0; 21)	**< .001**
A10 (mm)	9 (-23; 21)	4.5 (-2; 22)	**< .001**
MCF (mm)	8 (-24; 25)	4 (-5; 22)	**< .001**
α (degrees)	6 (-5; 178)	9 (-14; 178)	**< .001**

All values are shown as median (min-max), all tests show Mann Whitney U distribution testing, significant values in bold, n = number of patients with valid observations. Abbreviations: A5 = amplitude at 5 minutes, A10 = amplitude at 10 minutes, MCF = maximum clot formation, α = alpha angle.

Heparin resistance, which was defined as a failure to achieve an ACT of ≥ 450 seconds on the initial dose of heparin (300 IU.kg^-1^), was documented in both groups, with a total of 24 of 70 (34%) patients: 13 patients (42%) in the IE group and 11 (28%) in the non-IE group (p = .296). Patients with IE received an initial median dose of 336 IU.kg^-1^ (305–356 IU.kg^-1^), and a total dose of 558 IU.kg^-1^ (464–638 IU.kg^-1^) to achieve the target ACT of ≥450 s. Patients without IE received an initial median dose of 313 IU.kg^-1^ (307–330 IU.kg^-1^) and a total dose of 479 IU kg^-1^(463–527 IU.kg^-1^). These differences were not statistically significant (p = .087). All patients with heparin resistance received extra heparin, while none of them was given antithrombin-III concentrate or FFP.

**Fig 3:**
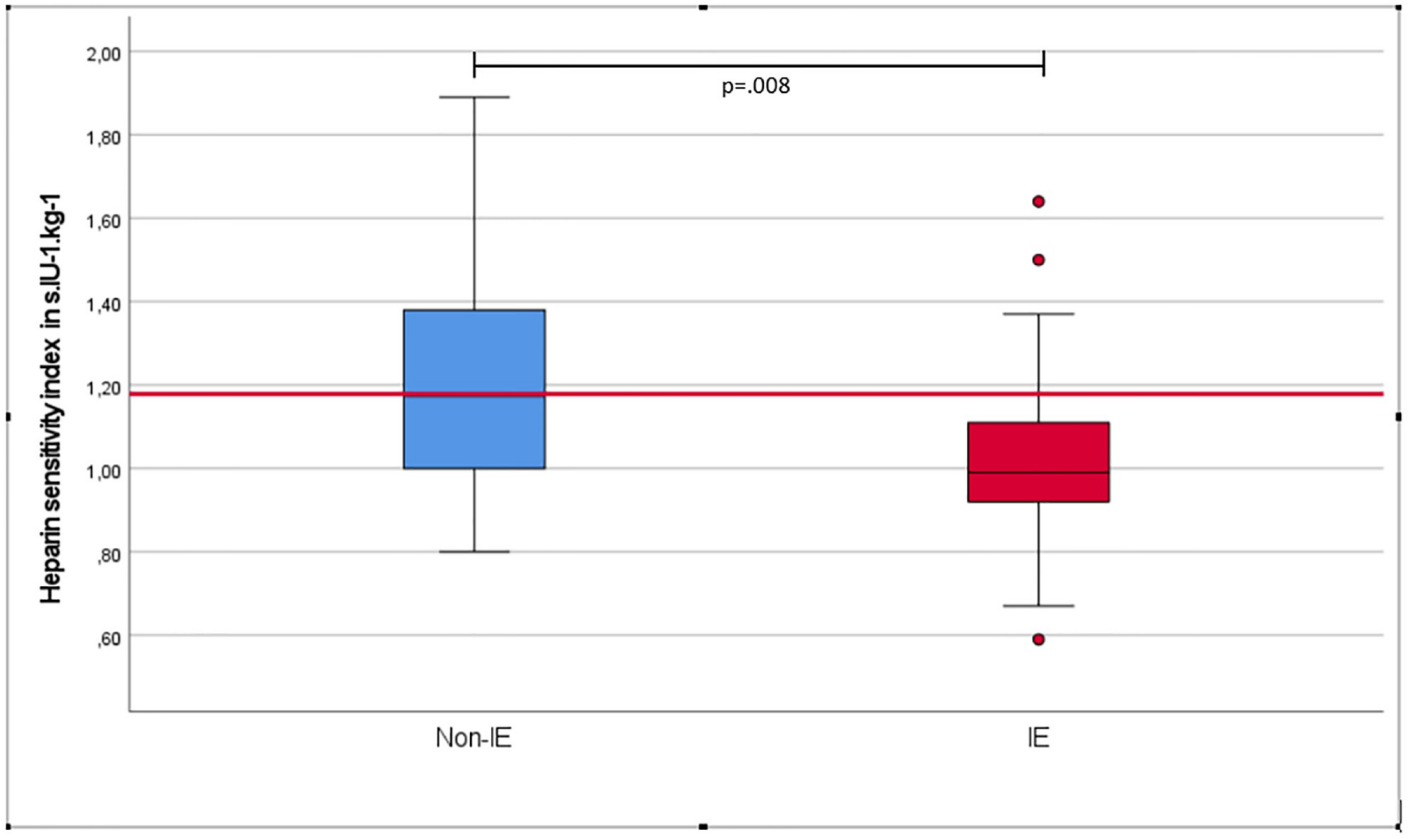
Heparin sensitivity index (HSI), comparing patients with and without infective endocarditis. The red line indicates the median HSI in patients without IE. Numbers indicate the median HIS of the respective group.

To assess heparin sensitivity, the HSI was calculated and compared between the groups. The non-IE group had a median index of 1.17 s.IU^-1^.kg^-1^ (min-max 0.80–1.89) and the IE group a median index of 0.99 s.IU^-1^.kg^-1^ (min-max 0.59–1.64), (U = 342.5, z = -2.64, p = .008) (Fig 3).

To investigate whether patients with heparin resistance showed distinct profiles in ROTEM measurements, we compared the ROTEM values between patients with and without heparin resistance (from both groups, IE and non-IE). Several clot firmness parameters in EXTEM and FIBTEM as well as CFT and α angle were significantly higher in patients with heparin resistance (Table 5).

**Table 5:** Preoperative ROTEM values in patients with and without heparin resistance.

ROTEM parameter	Heparin resistance (n = 24)	No heparin resistance (n = 46)	p-value
**EXTEM**			
CT	65 (53–108)	67 (38–123)	.753
A5	53 (31–69)	50 (38–70)	.167
A10	63 (40–77)	60 (49–77)	.130
CFT	62 (36–148)	73 (41–113)	**.040**
MCF	70 (51–83)	67 (56–79)	.260
α	80 (65–83)	76 (68–82)	**.011**
MCE	229 (104–474)	205 (128–387)	.266
AUC	6841 (8–8175)	6705 (5596–7767)	.638
**INTEM**			
CT	181 (130–313)	181 (114–283)	.939
A5	53 (27–76)	49 (25–66)	.100
A10	61 (37–81)	59 (45–73)	.122
CFT	57 (28–181)	63 (40–144)	.071
MCF	67 (49–83)	65 (51–77)	.216
α	79 (58–84)	77 (66–81)	**.039**
MCE	200 (94–493)	190 (104–327)	.249
AUC	6652 (4862–8213)	6494 (5186–7642)	.252
**FIBTEM**			
CT	59 (47–90)	60 (44–95)	.995
A5	22 (5–44)	18 (8–36)	**.029**
A10	24 (6–47)	20 (9–39)	**.041**
CFT	70 (4–382)	170 (45–1326)	**.004**
MCF	26 (6–51)	22 (9–43)	.066
α	80 (69–84)	77 (61–84)	**.004**
MCE	35 (6–104)	27 (10–77)	.052
AUC	2549 (560–5051)	2110 (891–4347)	.067

All values are shown as median (min-max), all tests show Mann Whitney U distribution testing, significant values in bold, n = number of patients with valid observations. Abbreviations: A5 = amplitude at 5 minutes, A10 = amplitude at 10 minutes, MCF = maximum clot formation, α = alpha angle.

When comparing the UDPB between the groups, patients with IE were more likely to experience moderate or severe bleeding than patients without IE. The odds associated with more severe perioperative bleeding in patients with IE was a factor 3.0 higher than in patients without IE (p = .018). Class 4 bleeding (massive) was not documented, because this requires the administration of rFVIIa concentrate, which was not the case in any patient in this study.

Postoperative blood loss in the first 12 and 24 hours was not significantly different between the groups (12 h: 340 ml in the IE group vs. 240 ml; 24 h: 400 ml vs. 350 ml, respectively). The proportion of patients with IE requiring a re-exploration was 25% vs 10% in the control group. More patients with IE underwent an early re-exploration (within one week) compared to patients without IE (p = .018).

**Fig 4:**
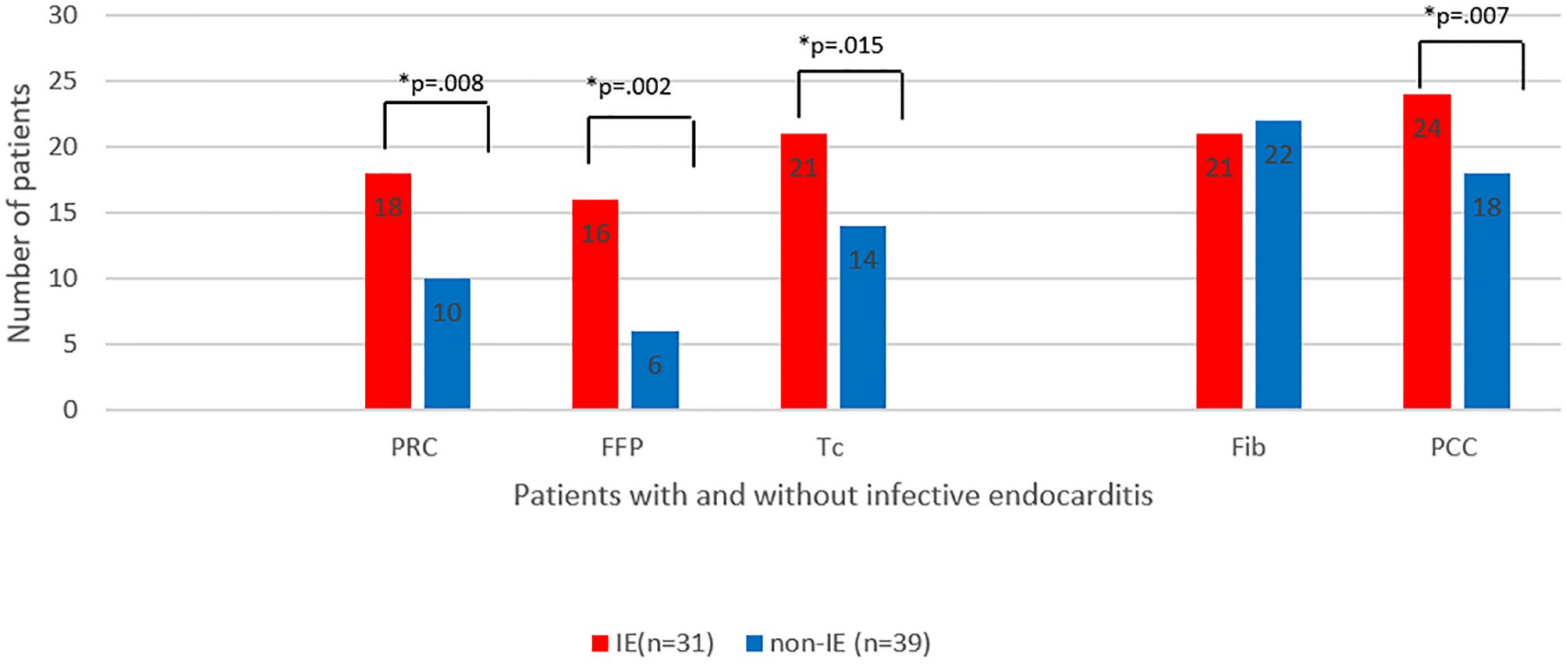
Number of patients receiving transfusion and coagulation factors in patients with and without infective endocarditis. *Indicates statistical significance. Abbreviations: RBC = packed red cells, FFP = fresh frozen plasma, Tc = thrombocytes, Fib = fibrinogen concentrate, PCC = prothrombin complex concentrate.

More patients with IE received RBC (p = .008), FFP (p = .002), platelets (p = .015) and prothrombin complex concentrate (PCC) (p = .007), while the number of patients who received fibrinogen concentrate was not different (Fig 4). All patients from both groups received tranexamic acid, whereby the dose did not differ between groups.

## Discussion

In the present study, we show for the first time, a full perioperative ROTEM profile in patients with IE undergoing cardiac valve surgery. With respect to the primary outcome, we show that IE causes a distinct coagulation pattern before, during and after surgery that is significantly different to patients without IE. These differences were observed in all ROTEM channels measured (EXTEM, INTEM and FIBTEM): Generally, a hypercoagulable state was noticed in patients with IE, reflected by higher clot firmness values and reduced clot formation time. Concomitantly, signs of consumption coagulopathy, characterized by prolonged clotting times in EXTEM were observed, indicating a deficiency of coagulation factors [14].

In previous studies, hypercoagulable patterns have been demonstrated in patients with IE, showing a reduced antithrombin III activity, enhanced platelet activity, and impaired fibrinolysis, eventually leading to thrombotic complications and heparin resistance [5, 18–20].

In a recent study on conservatively treated patients with IE [21], coagulation status was assessed by thromboelastography (TEG) and classical laboratory parameters. The authors found an increase in D-dimer and fibrinogen concentration in IE patients, while TEG parameters were not different to those from healthy volunteers. The exception was maximum amplitude (MA), which would correspond to MCF in ROTEM. In very severe cases, however, hypocoagulable profiles were documented (interpreted by the authors as progression into consumption coagulopathy), whereas patients developing thromboembolic complications displayed a decreased R parameter, which is analogous to clotting time in ROTEM. Thus, in general, our findings are similar to those by Koltsova et al., concerning hypercoagulopathic patterns in patients with IE [21]. The differences between patients with and without IE, however, were more pronounced in our study. This might be due to ROTEM being more sensitive to IE-related changes. Another reason may be the fact that we only investigated surgically treated patients, where the control group was patients undergoing similar cardiac valve surgery for other reasons, in contrast to the healthy volunteers used for validation.

Coagulopathy in patients with IE is likely to be multifactorial [4], especially in the perioperative period [10]. Hence, ROTEM analysis cannot assess the whole picture of coagulopathy in IE, such as platelet dysfunction. Platelet function analysis may be of benefit in future clinical studies.

The results from our ROTEM analysis display similarities to the results of patients with sepsis, in whom general hypercoagulability is also seen. However, patients with septic shock and DIC can also display hypocoagulability, which is associated with increased mortality [12, 22]. These similarities are not surprising as both IE and sepsis are systemic inflammatory diseases, and endocarditis can progress into sepsis and septic shock [23].

Similar to sepsis, DIC can occur in IE, with an incidence of up to 20%. Patients with IE who develop DIC have a significantly higher mortality and rate of recurrence of sepsis, than those without DIC. Outcome in these patients can be improved by early surgery [6], thus, it would be valuable to detect consumption coagulopathy as early as possible and to predict possible progression to DIC. In sepsis, DIC correlates with the ROTEM parameters CT, CFT, alpha angle and MCF of the EXTEM assay [24–26]. Whether laboratory measurements, such as these particular ROTEM parameters, assessed in an early state of disease could assist in predicting the development of DIC in patients with IE is unknown. The ability to predict DIC would be very valuable given that early surgery may improve outcome in those patients. In our study, only one patient developed DIC, so we are unable to make any assertions. Future studies on the suitability of ROTEM to predict the development of DIC should be encouraged.

During the course of the operation, clot firmness parameters decreased in all patients, as seen in the ROTEM assessed after declamping of the aorta. Likewise, clotting times increased, reflecting the development of coagulopathy in response to cardiopulmonary bypass, haemodilution and blood loss. Nevertheless, nearly all ROTEM variables were significantly different between patients with and without IE at this time point, reflecting that hypercoagulability is still present in patients with IE.

Interestingly, we found that after declamping, clot firmness values in the FIBTEM channel decreased more strongly (higher Δ FIBTEM) in the IE group compared to the non-IE group, although absolute values were still higher, and largely within the normal range. In a previous clinical study on ROTEM measurements in major orthopaedic surgery, Shin et al., found that the change rate (MCFFIB-C)) between the pre- and postoperative FIBTEM showed a significant correlation with intraoperative blood loss and red blood cell transfusion, with a cut-off value of ≥ 29% for MCFFIB-C [27]. Whether such a cut-off value might also be useful in cardiac surgery in general, or in patients undergoing valve surgery due to IE in particular, remains to be elucidated. Furthermore, the reasons why FIBTEM decreased more strongly in patients with IE remains elusive. Possibly, the coagulation system in IE is more susceptible to the deleterious effects of cardiopulmonary bypass due to the inflammatory state. However, this hypothesis will require further investigation in future studies.

When regarding secondary outcomes, patients with IE had a higher incidence of severe bleeding as assessed by the UDPB as well as more and earlier re-explorations. Furthermore, they received transfusions of RBC, FFP, thrombocytes and administration of PPC more frequently, which, concerning RBC transfusion, can partially be explained by a lower preoperative haemoglobin.

From current literature, it is not entirely clear, whether IE *per se* is a risk factor for increased transfusion of blood products in cardiac surgery. Only a very few studies on this topic have been published, most of which used a retrospective study design. In two large retrospective studies [28, 29], IE was identified as risk factor for massive blood transfusion, defined as more than four or five units of RBC on the day of surgery, respectively. Likewise, Ravn et al., showed that patients with IE undergoing valve surgery not only received more RBC, but also more FFP and platelet transfusions, compared to cardiac surgery for other reasons [30]. In contrast, Dahn et al., compared patients undergoing aortic valve replacement with or without IE, and found that patients with IE had a higher overall transfusion rate. After performing multivariable regression analysis, the authors concluded that IE was not an independent risk factor for transfusion, and that the characteristics associated with IE, such as anaemia and renal failure, accounted for the higher transfusion rate [31]. Furthermore, there is evidence to suggest that patients with IE are more likely to receive platelet transfusion than patients who do not have IE [32, 33].

The incidence of heparin resistance, defined as a failure to achieve the target ACT of ≥ 450 s after the initial dose of heparin, was higher in patients with IE (42% vs 29%), although this difference was not statistically significant. Patients with IE received a higher initial dose of heparin per kilogram on average, compared to patients without IE, probably due to the clinical experience of the attending anaesthetist. To correct for this effect, we assessed the HSI, which was significantly lower in the IE group, thus showing relative heparin insensitivity.

While the overall incidence of heparin resistance during cardiac surgery ranges from 4% to 26% (depending on the definition) [16], a recent retrospective study found a 31% incidence of HR in patients with IE, which is similar to our study. In this study, HSI was found to be significantly lower in patients with IE [9]. Thus, we supply further evidence that IE increases the resistance to heparin.

We showed that several ROTEM parameters were associated with the occurrence of heparin resistance and future studies should determine whether it might be possible to predict occurrence of heparin resistance by an initial ROTEM analysis. This may assist in preoperative preparation, e.g. for a higher dose or escalating doses of heparin, antithrombin-III concentrate or FFP to prevent insufficient anticoagulation before/during CPB.

In previous studies, certain ROTEM profiles were associated with pro-thrombotic complications such as in major non-cardiac surgery [34, 35] or Covid-19 [36]. The ability to predict thrombotic events more accurately would allow to prevent devastating complications such as stroke, and to assist in decision-making concerning timing of surgery [37].

The main limitation of our study is the exploratory nature, and the fact that we did not perform a sample size analysis. Nevertheless, we are confident that the data presented will be of great value for designing future studies in which the potential of ROTEM to predict complications in IE, or to evaluate the effectiveness of perioperative IE-specific ROTEM-driven transfusion algorithms is investigated. Furthermore, ROTEM analysis cannot account for all coagulation abnormalities, such as platelet dysfunction.

Given that perioperative ROTEM results in patients with IE differ significantly from patients without IE, it is conceivable that existing POCT-driven coagulation management algorithms designed for general cardiac surgery may need to be adjusted for patients with IE. This would individualize the perioperative care in these often high-risk patients undergoing cardiac valve surgery.

## Supporting information

S1 Table ROTEM parameters of IE and non-IE patients *after aortic declamping*. All values are shown as median (min-max), all tests show Mann Whitney U distribution testing, significant values in bold, n = number of patients with valid observations. Abbreviations: s = seconds, CT = clotting time, A5 = amplitude at 5 minutes, A10 = amplitude at 10 minutes, CFT = clot formation time, MCF = maximum clot formation, α = alpha angle, MCE = maximum clot elasticity, AUC = area under the first derivate curve. The CT and CFT are measured in seconds, alpha-angle is measured in degrees, MCF and A10 are measured in mm.
(DOCX)

S2 Table ROTEM parameters of IE and non-IE patients *at the end of surgery*. All values are shown as median (min-max), all tests show Mann Whitney U distribution testing, significant values in bold, n = number of patients with valid observations. Abbreviations: s = seconds, CT = clotting time, A5 = amplitude at 5 minutes, A10 = amplitude at 10 minutes, CFT = clot formation time, MCF = maximum clot formation, α = alpha angle, MCE = maximum clot elasticity, AUC = area under the first derivate curve. The CT and CFT are measured in seconds, alpha-angle is measured in degrees, MCF and A10 are measured in mm.
(DOCX)

S3 Table Lysis parameters of preoperative ROTEM of IE and non-IE patients. All values are shown as median (min-max), Mann Whitney U distribution testing performed, unable to compute a p-value, n = number of patients with valid observations.
(DOCX)

S4 Table Characteristics of infective agent in patients with endocarditis. ^1^Other staphylococci: Staphylococcus epidermidis, Staphylococcus lugdunensis. ^2^Other streptococci: Streptococcus agalactiae, Streptococcus dysgalactiae, Streptococcus gordonii, Streptococcus oralis, and Streptococcus pneumonia. ^3^Other agents: Abiotrophia defectiva, Aerococcus urinae, Aspergillus niger, Cutibacterium acnes (previously Proprionibacterium spp).
(DOCX)
