# Supplementary material for: Patients with infective endocarditis undergoing cardiac surgery have distinct ROTEM profiles and more bleeding complications compared to patients without infective endocarditis
Source: PLoS One. 2023 Apr 13;18(4):e0284329. doi: 10.1371/journal.pone.0284329 (PMC10101476; doi:10.1371/journal.pone.0284329)
Supplement: S1 Table — All values are shown as median (min-max), all tests show Mann Whitney U distribution testing, significant values in bold, n = number of patients with valid observations. Abbreviations: s = seconds, CT = clotting time, A5 = amplitude at 5 minutes, A10 = amplitude at 10 minutes, CFT = clot formation time, MCF = maximum clot formation, α = alpha angle, MCE = maximum clot elasticity, AUC = area under the first derivate curve. The CT and CFT are measured in seconds, alpha-angle is measured in degrees, MCF and A10 are measured in mm. (DOCX) [file pone.0284329.s001.docx]

**Supplementary Table 1.** ROTEM parameters of IE and non-IE patients *after aortic declamping*

| ROTEM parameter | |  | IE group | | non-IE group | p-value |
| --- | --- | --- | --- | --- | --- | --- |
| EXTEM | *n=29* | | | *n=39* | |  |
| CT (sec) | 112 (30-186) | | | 83 (55-181) | | **.024** |
| A5 (mm) | 45 (22-66) | | | 38 (20-53) | | **.002** |
| A10 (mm) | 57 (31-72) | | | 49 (29-64) | | **.002** |
| CFT (sec) | 75 (6-255) | | | 110 (62-293) | | **.001** |
| MCF (mm) | 66 (28-78) | | | 61 (44-72) | | **.005** |
| Α (degrees) | 75 (49-89) | | | 68 (49-78) | | **<.001** |
| MCE | 193 (40-364) | | | 154 (78-261) | | **.003** |
| AUC | 6569 (2863-7579) | | | 6039 (4398-7275) | | **.003** |
| Lysis 30 min | 100 (100) | | | 100 (100) | | **-** |
| Lysis 45 min | 100 (95-100) | | | 100 (99-100) | | **.049** |
| Lysis 60 min | 99 (89-100) | | | 99 (94-100) | | .764 |
| Max Lysis | 2 (0-100) | | | 4 (0-100) | | .856 |
| Clot Lysis rate | 9 (8-10) | | | 8 (4-90) | | .633 |
|  |  | | |  | |  |
| FIBTEM | *n=29* | | | *n=39* | |  |
| CT (sec) | 106 (21-183) | | | 77 (57-285) | | **<.001** |
| A5 (mm) | 17 (9-41) | | | 11 (2-21) | | **<.001** |
| A10 (mm) | 19 (10-43) | | | 12 (3-22) | | **<.001** |
| CFT (sec) | 314 (58-2238) | | | 790 (181-1990) | | .199 |
| MCF (mm) | 22 (11-45) | | | 13 (5-27) | | **<.001** |
| Α (degrees) | 73 (53-83) | | | 69 (52-85) | | .201 |
| MCE | 15 (12-81) | | | 15 (5-37) | | **<.001** |
| AUC | 2190 (1068-4408) | | | 1295 (502-2687) | | **<.001** |
| Lysis 30 min | 100 (100) | | | 100 (100) | | **-** |
| Lysis 45 min | 100 (100) | | | 100 (98-100) | | .368 |
| Lysis 60 min | 100 (99-100) | | | 100 (96-100) | | .752 |
| Max Lysis | 9.8 (29) | | | 9.5 (26) | | .366 |
| Clot Lysis rate | 6.5 (4-9) | | | 9 (6-44) | | .262 |
|  |  | | |  | |  |
| HEPTEM | *n=29* | | | *n=39* | |  |
| CT (sec) | 272 (183-936) | | | 242 (187-359) | | **.042** |
| A5 (mm) | 40 (7-56) | | | 38 (7-57) | | .544 |
| A10 (mm) | 51 (13-65) | | | 48 (11-65) | | .231 |
| CFT (sec) | 95 (58-1031) | | | 105 (46-1520) | | .624 |
| MCF (mm) | 61 (35-71) | | | 58 (25-73) | | .268 |
| Α (degrees) | 72 (16-78) | | | 69 (14-80) | | .471 |
| MCE | 158 (79-250) | | | 140 (34-264) | | .123 |
| AUC | 6044 (3564-7180) | | | 5847 (2558-7231) | | .377 |
| Lysis 30 min | 100 (100) | | | 100 (100) | | - |
| Lysis 45 min | 100 (94-100) | | | 100 (97-100) | | .208 |
| Lysis 60 min | 98 (89-100) | | | 98 (94-100) | | .938 |
| Max Lysis | 3.5 (0-100) | | | 4 (0-100) | | .990 |
| Clot Lysis rate | 7 (4-8) | | | 8 (3-11) | | .556 |
