# Supplementary material for: Patients with infective endocarditis undergoing cardiac surgery have distinct ROTEM profiles and more bleeding complications compared to patients without infective endocarditis
Source: PLoS One. 2023 Apr 13;18(4):e0284329. doi: 10.1371/journal.pone.0284329 (PMC10101476; doi:10.1371/journal.pone.0284329)
Supplement: S2 Table — All values are shown as median (min-max), all tests show Mann Whitney U distribution testing, significant values in bold, n = number of patients with valid observations. Abbreviations: s = seconds, CT = clotting time, A5 = amplitude at 5 minutes, A10 = amplitude at 10 minutes, CFT = clot formation time, MCF = maximum clot formation, α = alpha angle, MCE = maximum clot elasticity, AUC = area under the first derivate curve. The CT and CFT are measured in seconds, alpha-angle is measured in degrees, MCF and A10 are measured in mm. (DOCX) [file pone.0284329.s002.docx]

**Supplementary Table 2.** ROTEM parameters of IE and non-IE patients *at the end of surgery*

| ROTEM parameter | | | IE group (n=31) | non-IE group (n=39) | p-value |
| --- | --- | --- | --- | --- | --- |
|  | | |  |  |  |
| EXTEM | | | *n=30* | *n=39* |  |
| CT (sec) | | | 71 (60-774) | 70 (53-387) | .194 |
| A5 (mm) | | | 45 (7-58) | 38 (27-58) | **<.001** |
| A10 (mm) | | | 55 (12-72) | 48 (39-67) | **<.001** |
| CFT (sec) | | | 19 (41-809) | 116 (46-162) | **<.001** |
| MCF (mm) | | | 65 (50-78) | 59 (50-73) | **.028** |
| Α (degrees) | | | 75 (20-82) | 70 (59-80) | **<.001** |
| MCE | | | 180 (40-274) | 148 (0-274) | **.001** |
| AUC | | | 6388 (2938-7340) | 5933 (4829-7271) | **.001** |
| Lysis 30 min | | | 100 (100) | 100 (100) | **-** |
| Lysis 45 min | | | 100 (96-100) | 100 (97-100) | .201 |
| Lysis 60 min | | | 99 (9-100) | 99 (94-100) | .786 |
| Max Lysis | | | 4 (0-100) | 4 (0-100) | .995 |
| Clot Lysis rate | | | 3 (2-4) | 4 (3-5) | **.031** |
|  | | |  |  |  |
| INTEM | | *n=31* | | *n=36* |  |
| CT (sec) | | 231 (158-1135) | | 227 (144-377) | .606 |
| A5 (mm) | | 45 (5-64) | | 36 (29-58) | **.001** |
| A10 (mm) | | 55 (12-72) | | 48 (39-67) | **.001** |
| CFT (sec) | | 79 (41-809) | | 116 (46-162) | **.001** |
| MCF (mm) | | 65 (20-78) | | 59 (50-73) | **.003** |
| Α (degrees) | | 75 (20-82) | | 70 (59-80) | **<.001** |
| MCE | | 183 (99-346) | | 141 (100-276) | **.002** |
| AUC | | 6417 (4980-7710) | | 5831 (4992-7290) | **.004** |
| Lysis 30 min | | 100 (100) | | 100 (100) | **-** |
| Lysis 45 min | | (97-100) | | (96-100) | .517 |
| Lysis 60 min | | 99(9-100) | | 100 (94-100) | .663 |
| Max Lysis | | 4 (0-100) | | 4 (0-100) | .366 |
| Clot Lysis rate | | 3 (2-4) | | 4 (3-5) | .223 |
|  | |  | |  |  |
| FIBTEM | *n=30* | | | *n=37* |  |
| CT (sec) | 65 (45-87) | | | 61 (48-93) | .174 |
| A5 (mm) | 18 (12-40) | | | 11 (7-21) | **<.001** |
| A10 (mm) | 19 (13-43) | | | 12 (8-25) | **<.001** |
| CFT (sec) | 255 (42-4389) | | | 880 (238-3507) | **.008** |
| MCF (mm) | 21 (14-45) | | | 14 (8-31) | **<.001** |
| Α (degrees) | 77 (60-85) | | | 72 (53-84) | **<.001** |
| MCE | 27 (17-81) | | | 17 (9-44) | **<.001** |
| AUC | 2120 (1426-4428) | | | 1416 (833-3042) | **<.001** |
| Lysis 30 min | 100 (96-1S00) | | | 100 (100) | .267 |
| Lysis 45 min | 100 (93-100) | | | 100 (94-100) | .779 |
| Lysis 60 min | 100 (85-100) | | | 100 (96-100) | .729 |
| Max Lysis | 0 (0-100) | | | 0 (0-100) | .907 |
| Clot Lysis rate | 8 (7-11) | | | 6 (5-13) | .151 |
|  |  | | |  |  |
| HEPTEM | *n=29* | | | *n=36* |  |
| CT (sec) | 214 (52-367) | | | 222 (143-388) | .668 |
| A5 (mm) | 44 (29-59) | | | 37 (23-54) | **.001** |
| A10 (mm) | 54(43-69) | | | 47 (36-64) | **.001** |
| CFT (sec) | 82 (48-188) | | | 107 (55-249) | **.005** |
| MCF (mm) | 63 (53-73) | | | 59 (50-72) | **.001** |
| Α (degrees) | 75 (55-80) | | | 72 (48-79) | **.006** |
| MCE | 169 (111-265) | | | 146 (101-255) | **.007** |
| AUC | 6252 (5331-7231) | | | 5905 (5026-7146) | **.001** |
| Lysis 30 min | 100 (98-100) | | | 100 (10-100) | .895 |
| Lysis 45 min | 100 (94-999) | | | 100 (97-100) | .151 |
| Lysis 60 min | 98 (92-100) | | | 98 (93-100) | .973 |
| Max Lysis | 5 (0-100) | | | 5 (0-100) | .671 |
| Clot Lysis rate | 7 (6-8) | | | 7 (6-365) | **.019** |
