# Supplementary material for: Patients with infective endocarditis undergoing cardiac surgery have distinct ROTEM profiles and more bleeding complications compared to patients without infective endocarditis
Source: PLoS One. 2023 Apr 13;18(4):e0284329. doi: 10.1371/journal.pone.0284329 (PMC10101476; doi:10.1371/journal.pone.0284329)
Supplement: S3 Table — All values are shown as median (min-max), Mann Whitney U distribution testing performed, unable to compute a p-value, n = number of patients with valid observations. (DOCX) [file pone.0284329.s003.docx]

**Supplementary Table 3.** Lysis parameters of preoperative ROTEM of IE and non-IE patients

| EXTEM | IE (*n=28)* | Non IE (*n=39)* | p-value |
| --- | --- | --- | --- |
| Lysis 30 min | 100 (98-100) | 100 (98-100) | **-** |
| Lysis 45 min | 98 (95-100) | 98 (94-100) | **-** |
| Lysis 60 min | 95 (91-99) | 95 (89-100) | **-** |
| Max Lysis | 10.5 (6.5-11.0)^ | 11 (8.0-13.0)^ | **-** |
| Clot Lysis rate | 8 (8-9) | 9 (7-10) | **-** |

All values are shown as median (min-max), Mann Whitney U distribution testing performed, unable to compute a p-value, n=number of patients with valid observations.
