# Supplementary material for: Patients with infective endocarditis undergoing cardiac surgery have distinct ROTEM profiles and more bleeding complications compared to patients without infective endocarditis
Source: PLoS One. 2023 Apr 13;18(4):e0284329. doi: 10.1371/journal.pone.0284329 (PMC10101476; doi:10.1371/journal.pone.0284329)
Supplement: S4 Table — 1Other staphylococci: Staphylococcus epidermidis, Staphylococcus lugdunensis. 2Other streptococci: Streptococcus agalactiae, Streptococcus dysgalactiae, Streptococcus gordonii, Streptococcus oralis, and Streptococcus pneumonia. 3Other agents: Abiotrophia defectiva, Aerococcus urinae, Aspergillus niger, Cutibacterium acnes (previously Proprionibacterium spp). (DOCX) [file pone.0284329.s004.docx]

**Supplementary Table 4.** Characteristics of infective agent in patients with endocarditis

| Infective agent n (%) | Endocarditis (n=31) |
| --- | --- |
| Staphylococci |  |
| *staphylococcus aureus* | 2 (6) |
| Other staphylococci^1^ | 4(13) |
| Streptococci: |  |
| Viridans streptococci |  |
| *Streptococcus salivarius* | 1(3) |
| *Streptococcus sanguinis* | 1(3) |
| Streptococcus bovis |  |
| *Streptococcus gallolyticus* | 3(10) |
| Other streptococci^2^ | 8(26) |
| Enterococci *(E. faecalis)* | 2(6) |
| Other agents^3^ | 5(16) |
| Unknown agent | 5(16) |

^1^Other staphylococci: Staphylococcus epidermidis, Staphylococcus lugdunensis

^2^Other streptococci: Streptococcus agalactiae, Streptococcus dysgalactiae, Streptococcus gordonii, Streptococcus oralis, and Streptococcus pneumonia

^3^Other agents: Abiotrophia defectiva, Aerococcus urinae, Aspergillus niger, Cutibacterium acnes (previously Proprionibacterium spp)
